# Pediatric pulmonary multisystem langerhans cell histiocytosis: does lung lesion severity affect the outcome?

**DOI:** 10.1186/s13023-023-02970-5

**Published:** 2023-11-17

**Authors:** Mohamed Sedky, Seham Gohar, Sonia Ahmed, Iman Zaky, Asmaa Salama, Omayma Hassanein, Eslam Maher, Alaa ElHaddad

**Affiliations:** 1 Department of Pediatric Oncology, Children Cancer Hospital Egypt 57357 1 (11617), Sekket Al-Imam St., Al Sayyeda Zeinab, Cairo, 11617 Egypt; 2 Department of Pediatrics, National Research Centre, Cairo, Egypt; 3 Department of Pediatric Oncology National Cancer Institute, Cairo University, Cairo, Egypt; 4 Department of Radiology, Children Cancer Hospital Egypt 57357 (11617), Cairo, Egypt; 5 Department of Radiology National Cancer Institute, Cairo University, Cairo, Egypt; 6 Department of Pathology, Children’s Cancer Hospital 57357 (11617), Cairo, Egypt; 7 Department of Pathology National Cancer Institute, Cairo University, Cairo, Egypt; 8 Department of Clinical Research, Children’s Cancer Hospital 57357 (11617), Cairo, Egypt

**Keywords:** Pulmonary, LCH, Multisystem, Survival, RO−, RO+, Lung lesions, Severity

## Abstract

**Background:**

The pediatric pulmonary multisystem Langerhans cell histiocytosis (PPM LCH) is associated with either low risk or high risk organ(s). The nodulo-cystic lung lesions although pathognomonic, yet are very variable in severity and remain a source of controversy in certifying pulmonary LCH diagnosis. The study aimed to examine the prognostic value of clinical respiratory manifestations and radiological lung lesions severity. This is through associating a CT chest triad of bilateral, extensive and diffuse lesions. It is a retrospective study of 350 LCH patients who received systemic treatment at Children’s Cancer Hospital Egypt during the period from 2007 to 2020.

**Results:**

Sixty-seven patients (67/350–19.1%) had PPM LCH at presentation. Severe lung lesions were present in 24 of them. The median follow-up period was 61 months (IQR: 3.4–8.3). The 5-year overall survival (OS) and event free survival (EFS) was 89% and 56.6% respectively. The EFS, for severe radiological lesions triad was 38% ± 20.7 versus 66% ± 16.2 for non-severe lesions triad *p* 0.002, while for presence of chest X-ray changes 27% ± 22.344 versus absence of chest X ray changes 66% ± 14.7 *p* 0.001, for clinical respiratory manifestations 13% ± 13.9 versus none 62% ± 22.9 *p* < 0.001, for RO− with severe lung lesions 47% ± 30.4 versus RO− without severe lung lesions 69% ± 5.9 *p* 0.04. There was a tendency for the independent prognostic impact of severe lung involvement; aHR = 1.7 (95% CI 0.92–3.13, *p* = 0.09).

**Conclusion:**

Although the lung is a low -risk organ per se in LCH, our study demonstrates a non negligeable prognostic impact of severe lung involvement in the risk stratification of pediatric LCH. This warrants further study and external validation.

**Supplementary Information:**

The online version contains supplementary material available at 10.1186/s13023-023-02970-5.

## Introduction

Langerhans cell histiocytosis LCH has become an inflammatory myeloid neoplasm [1–3]. It is a heterogeneous disease that can affect a single or multisystem with management ranging from observation to intensive therapy; thus tailoring treatment according to risk stratification [4].This is related to involvement of high risk organ(s) RO+ or low risk organ(s) RO- LCH [5–7]. The lung was part of RO+ group until 2012 when it has been excluded as Ronceray et al. showed that it is not an independent cause for mortality [8]. Contrarily to the reactionary adult form related to tobacco smoking, the pediatric pulmonary multisystem PPM LCH is a clonal neoplastic disease diagnosed clinico radiologically [9–11]. Clinical diagnosis might be undermined by lacking of respiratory manifestations in a good number of cases [12, 13]. Radiologically, plain chest X-ray (CXR) is used to show honey comb lesions, which might be absent in early cases. However, the pathognomonic nodulocystic condensations is better demonstrated by the usage of low dose high resolution chest CT scan [15–17]. Lung involvement at diagnosis is subjected to the physician perception of the disease; either by restricting the diagnosis to severe bilateral nodulocystic lesions [18] or confirming wrongly LCH lung involvement through isolated nonspecific pneumopathies [19]. This leads to inaccurate stratification of patients with subsequent under or over treatment. Such observations raised a question: Does lung lesion severity affect the outcome? We retrospectively analyzed PPM LCH patients presenting with typical chest high resolution CT nodules and cysts with or without clinical respiratory manifestations.

By standardizing specific clinical and radiological manifestations, the study aimed to examine the prognostic value of lung lesions severity in a single center large cohort.

### Patients and methods

During the period from June 2007 to the end December 2020, 425 de novo LCH patients were diagnosed at Children’s Cancer Hospital Egypt 57357. Seventy-five patients out of 425, were excluded from the study because they did not receive systemic treatment as they were unisystem unifocal LCH. This study is a retrospective analysis of 350 patients who received systemic chemotherapy with a median follow-up period of 61 months (0.8–176). Of them, we analyzed sixty seven consecutive PPM LCH patients, not related to tobacco smoking with lung involvement associated with either (RO−) n = 47 or RO+ n = 20.

*Data Collection and diagnosis *Electronic medical records were reviewed, data were collected and analyzed after the approval of the scientific and medical advisory committee (SMAC) as well as the Institutional Review Board (IRB). All patients were evaluated with comprehensive history and physical examination. Diagnosis was confirmed by a proven biopsy taken mainly from the most accessible and representative site. High resolution chest CT scan and plain chest X-ray were done to all patients. They were stratified according to the Histiocyte Society (HS) into those Low risk (RO−) with Single-system (SS)-unifocal (USUF)/multifocal (USMF)-or Multisystem (MS) LCH involving two or more organs (MSRO−). Otherwise, those High risk (RO+) with “risk organs” including the hematopoietic system, liver and spleen (MSRO+) [4, 20–23].

*Lung involvement* is radiologically defined by the presence of pathognomonic honey comb on plain x ray or nodulocystic lesions on high resolution chest CT scan [18, 24]. Clinically, the criteria of respiratory manifestations were extrapolated from the Friedmann classification of respiratory distress ranging from no respiratory complaint, eupnea (stage I) to subjective respiratory complaint, slight tachypnea (stage II) to moderate respiratory distress retractions, moderate tachypnea (stage III) to severe respiratory distress retractions, cyanoses, delirium, decreased consciousness, respiratory arrest (stage IV) [25]. In our study, radiological lung lesions severity was considered if a CT scan radiological triad was fulfilled. This included a triad of lesions that were (1) bilateral, (2) diffuse with pathognomonic nodules or cysts occupying each lobe with more than one segment per lobe and (3) extensive with innumerable nodules/cysts or pneumothorax. On the other hand, non-severe lung involvement was defined if the triad was not fulfilled.

*Treatment* During the period from mid-2007 till end 2011, the lung was part of RO+ group and patients were treated accordingly as MSRO+ with the LCH III protocol including: Induction I (initial 6 weeks) of oral Prednisone (PRED) 40 mg/m^2^/d, associated with weekly intravenous vinblastine (VBL) 6 mg/m^2^/d. Induction II (further 6 weeks) similar to Induction I but with day 1–3 weekly (PRED). Intermediate dose methotrexate (ID MTX) 500 mg/m^2^ every other week was added to both inductions. This was followed by one year continuation treatment including, 6 mercaptopurine (6MP) 50 mg/m^2^ daily and oral (MTX) 20 mg/week [4]. After 2011, the lung has been considered a RO− organ and patients shifted to the LCH IV excluding ID MTX from induction. This was followed by continuation treatment of VBL/PRED or Vincristine/Aracytine/PRED /6MP/MTX whether the lung was associated to RO− or RO+ respectively [20].

*Disease response to first line treatment* was assessed by the end of induction phase, as no active disease (NAD), active disease better (ADB), active disease intermediate (ADI) and active disease worse (ADW) [20, 26, 27] *Radiological response of lung lesions* was assessed at end of induction and at last follow up as progressed or stationary or regressed or cleared nodulo-cystic lesions.

*Failure of treatment *Indicators were either disease progression (DP) or reactivation (REA). Disease progression was recorded, if the patient showed progressed lesions during induction phase or failed to achieve better status (NAD or ADB) by the end of induction. Reactivation was recorded if the patient showed progressive lesions after having achieved better status by the end of the induction phase [20, 26, 27].

*Prognostic factors *in the lung involvement cohort included the age group, gender, disease risk stratification, and radiological plain CXR changes. The same was used for lung lesions severity whether clinical in the form of respiratory manifestations or radiological through a triad of bilateral, diffuse and extensive lesions and each apart. This radiological lung involvement and its triad of severity were tested for their impact on survival in RO− or RO+ (Hemopoietic or hepatic or splenic).

### Collection and statistical analysis

Kaplan–Meier analysis was used to estimate 5-year survival; overall survival (OS) was calculated from date of diagnosis until date of last follow-up or date of death, and event free survival (EFS) from date of diagnosis until date of REA, DP, last follow up or death. The main risk factors studied were binary variables: Age group, liver, spleen, hematopoietic system, and lung involvement. Age was dichotomized at 2 years similar to the pediatric LCH literature. Log rank tested the impact of different risk factors on survival. Lung involvement was collected as three strata: No lung involvement, no severe lung involvement, or severe lung involvement. Since the first two strata were almost identical with regards to their survival experience, they were recoded into the same stratum for the multivariable regression. EFS was modeled by Cox regression using the variables of interest, and adjusted Hazard Ratios and associated 95% confidence intervals were calculated. Model fit, interaction, discrimination, and calibration were evaluated. Proportionality of hazards were inspected graphically via Schoenfeld residuals. All tests are two-sided. Analysis was conducted using R version 4.1.2 and IBM SPSS statistics 22.0. *P*-values ≤ 0.05 were indicative of statistical significance and, tendency to be statistically significant if between 0.05 and 0.1.

## Results

This cohort included 350 LCH patients (M 207/F 143) who received systemic treatment. Patients less than 2 years of age were 102 (29%). Liver involvement was present in 127 (36%), spleen in 43 (12%), and hematopoietic system in 42 (12%). At initial presentation, 67 consecutive non tobacco smokers PPM LCH patients were included. They represented 16% of the whole population (67/425) and 19% of those receiving systemic treatment (67/350). Lung involvement was associated with high-risk organs (MSRO+) in 20 patients, while RO− in 47 patients. Of them, 37 with multisystem (MSRO−) and 10 with single system (USMF n = 6, USUF n = 4). The details of clinical radiological lung characteristics and outcome are shown in Additional file 1: Table S1. Seven patients were treated according to LCH III protocol, where the lung was considered the only high-risk organ in one patient (UPN 31) and associated with other RO+ in 6 patients (UPN 1, 2, 3, 4, 5, and 6) Additional file 1: Table S1. Forty-three patients were of the age above 2 years with a median age 2.7 y (0.4–17). Open lung biopsy was done once as no other accessible site could be provided.

### Clinical radiological characteristics

Out of 67 PPMLCH patients, significant clinical respiratory manifestations were present in 8 patients. CT scan was positive for pathognomonic nodulocystic LCH lung lesions in all patients while plain CXR changes were present in only 15 patients (22%). Solitary tiny nodule was considered a lung involvement associated with 2 RO+ and 17 RO−. Of them, USUF and USMF was the case in 2 patients respectively. The radiological triad of severe lesions (diffuse, extensive and bilateral), was associated with 13 MSRO− out of 37 and 11 MSRO+ out of 20 patients (Additional file 1: Table S1).

### Outcome

**Fig. 1:**
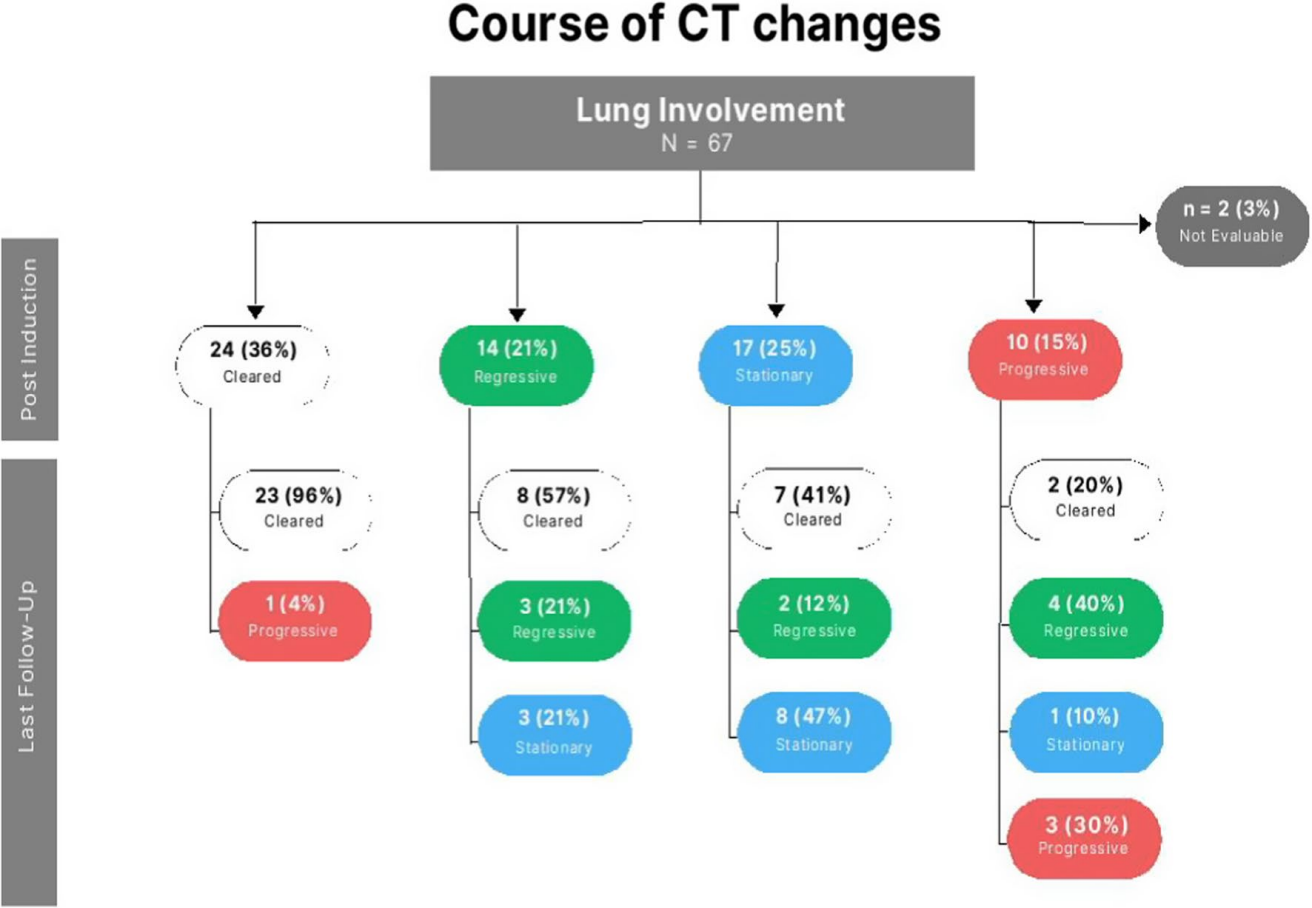
Course CT lung

Twenty-three PPMLCH patients failed 1st line treatment. Of them 10 progressed their disease (DP) at the end of induction and 13 showed reactivation (REA) afterwards. The kinetics of radiological lung lesions showed that 49 out of 67 patients cleared or regressed their lesions by their last follow up. The course of CT lung changes is shown in Fig. 1. Eight patients (12%), all in RO+ group, died. Of them, 4 patients had lung lesions progression at the time of death (2 as a part of multisystem failure and 2 exclusively due to lung disease progression). Otherwise, the remaining 4 patients died of other multisystem failure. The 5-year OS was 89% CI 7.84 and EFS 56.6% CI 12.936 (Additional file 1: Table S1).

### Factors affecting survival

By univariate analysis of the 67 patients, the age group less than 2 years showed a statistically significant lower OS. Otherwise, there was no impact of gender on survival. There was a statistically significant lesser OS 57% with RO+ vs 100% with RO-*p* < 0.001 and EFS 35% with RO+ vs 65% with RO− *p* 0.001. This was confirmed with risk subgrouping into MSRO+ , MSRO-, USUF, USMF. The factors affecting survival in lung LCH are shown in Table 1.

**Table 1:** Factors affecting survival in lung LCH

Event	N	%	OS (%)	*p*	CI	EFS (%)	*p*	CI
*Gender*								
F	26	39	88	0.99	± 19.9	65.8	0.389	± 13.132
M	41	61	51		± 16.8	89.7		± 9.604
Age group below	24	36	70	0.001	± 19	44	0.032	± 20
Age group above 2-year age	43	64	100			63		± 17
Lung with RO +	20	30	57	0	± 23	35	0.001	± 21
Lung with RO-	47	70	100			65		± 16
*Risk stratification*								
MS RO +	20	30	63.8	0	± 21.6	35	0.007	± 20.9
MS RO-	37	55	100			63		± 18.2
USMF	6	9	100			60		± 42.9
USUF	4	6	100			100		
Clinical respiratory manifestations	8	12	91	0.2	± 7.6	12.5	0	± 13.9
No respiratory	59	88	75		± 29.9	62.4		± 22.9
Radiological triad of lung severity severe	24	36	78	0.088	± 17	38	0.002	± 20.7
Mild	43	64	95		± 6.46	66		± 16.2
Radiological diffuse lesions	38	57	83.6	0.067	± 12.15	42.4	0.008	± 17.0
Radiological localized lesions	29	43	96		± 7.056-	75.9		± 17.4
Radiological extensive lesions	26	39	79.6	0.128	± 16.0	43.3	0.013	± 19.9
Radiological mild lesions	41	61	94.9		± 6.86	64.6		± 16.8
Bilateral radiological	44	66	85.7	0.198	± 10.78	49.9	0.103	± 16.268
Unilateral radiological	23	34	95.2		± 9.016	69.8		± 20.58
Chest X ray changes	15	22	80	0.317	± 20.18	26.7	0.001	± 22.344
No Chest X ray changes	52	78	91.4		± 8.036	65.7		± 14.7

CI confidence interval, *EFS* event free survival, *F* female, *M* male, MSRO+ multisystem high risk organ, MSRO− multisystem low risk organ, *N* number, *OS* overall survival, *p* value, *USMF* unisystem multifocal, *USUF* unisystem unifocal. Underline is to highlight significant p values

### Survival and clinical radiological lung involvement

In those 67 patients, there was a significant lesser EFS 12% with clinical respiratory manifestations vs 62% without *p* < 0.001. Lesser EFS 38% with radiological triad of diffuse, extensive and bilateral lesions was observed in comparison to no triad 66% *p* 0.007. This is confirmed statistically when each radiological diffuse or extensive was tested alone. Moreover, EFS with presence of CXR changes was 27% in relation to 66% for absence of CXR changes *p* 0.002 Table 1.

### Radiological lung lesions severity and risk stratification

**Fig. 2:**
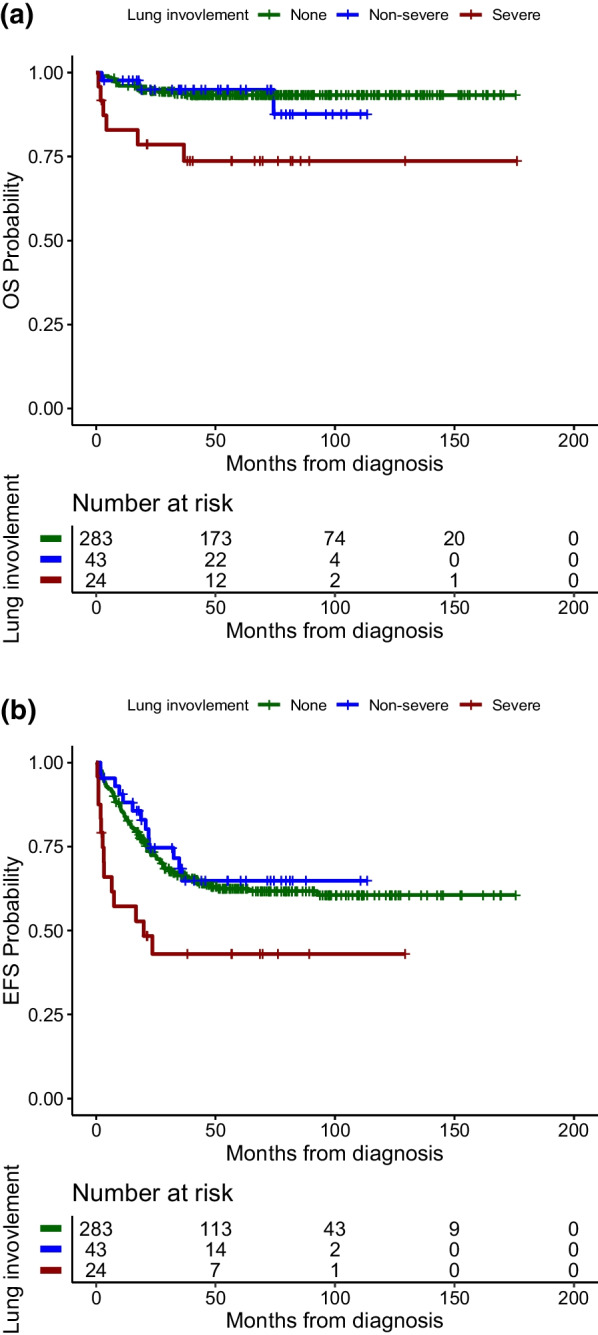
**a** OS of lung severity whole population. **b** EFS of lung severity whole population

**Fig. 3:**
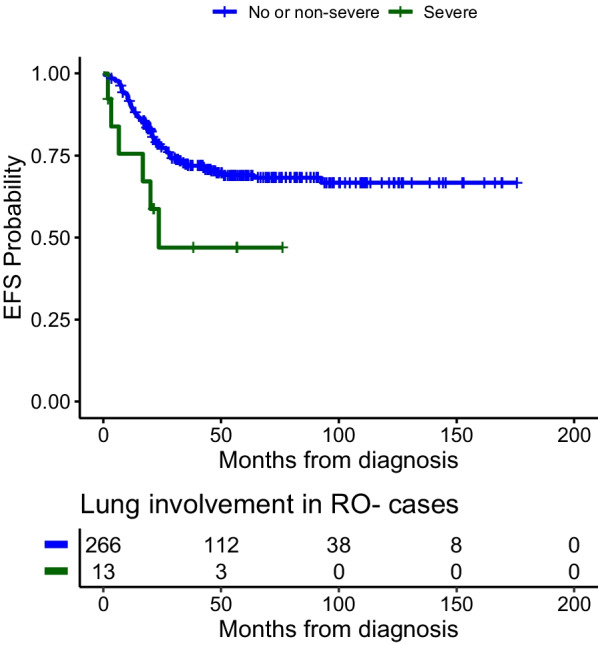
EFS of lung severity in RO−

With 350 patients (incorporating both RO+ and RO− groups) receiving systemic treatment, there was a statistically significant lesser OS and EFS when radiological severe lung lesions triad was involved. The OS and EFS of whole lung population are shown in Fig. 2a, b. In the RO− group, lesser EFS was 47% with radiological severe lesions triad vs 69% in non-severe lung lesions *p* 0.04. The EFS of lung severity in RO− is shown in Fig. 3. When considering RO+ , although there was no statistically significant lesser survival in severe lung lesions association in general, hepatic involvement and splenomegaly each was responsible of statistically significant lesser OS when associated with severe lung lesions. The stratification and radiological severe lung lesions effect on survival is shown in Table 2.

**Table 2:** Stratification and radiological severe lung lesions effect on survival

Event	N	%	OS (%)	*P*	CI	EFS (%)	*P*	CI (%)
LCH total	350							
Severe lung	24	7	73.3	0.001	57.4–94.3%	43	0.011	26.6–69.6
No severe lung	43	13	95		88.4–100%	64.8		50.8–82.7
No lung involvement	283	81	93.6		90.4–96.4%	62.5		56.8–68.7
RO−	279							
With severe lung	13	4	100	0.83	NA	47	0.04	16.6–77.4
With no severe lung	266	76	99.6		99.8–100%	69		63.1–74.9
RO+	71							
With severe lung	11	15	45.5	0.12	16.1–74.9%	36.4	0.44	8–64.8
With no severe lung	60	85	67		54.9–79.2%	35.8		23.3–48.3
Liver	127							
Severe lung	11/24	46	46	< 0.001	23.8–86.8%	27.3	0.003	10.4–71.6
No Severe lung	116/326	36	85.2		78.8–92.2%	49.8		41.1–60.4
Splenomegaly	43							
Severe lung	5/24	21	20	0.009	3.5–100%	20	0.2	3.5–100
No severe lung	38/326	12	61.8		48–79.8%	31		18.7–50.8
Hemopoietic system	42							
Severe lung	8/24	33	43.8	0.09	18.9–100%	43.8	0.8	18.9–100
No severe lung	34/326	10	70.1		56.1–87.5%	29.1		17.2–49.4

CI confidence interval, *EFS* event free survival, *N* number, *OS* overall survival, *p* value, RO− low risk organs, RO+ high risk organs. Underline is to highlight significant p values

In a multivariable model, the adjusted hazard ratio (aHR) for severe lung involvement was the largest, corresponding to 1.7 (95% CI 0.92–3.13, *p* = 0.09). Age group < 2 years old was the most important (positive) prognostic factor (aHR = 0.44, 95% CI 0.30–0.64, *p* < 0.001). Liver involvement, but not hematopoietic system *p* = 0.79 or spleen involvement *p* = 0.14, was an independent prognostic factor; aHR = 1.59 (95% CI 1.05–2.42, *p* = 0.03). The model showed good discrimination and calibration. The Multivariable cox regression for factors predictive of event-free survival are shown in Table 3.

**Table 3:** Multivariable cox regression for factors predictive of event-free survival

Characteristic	n (%)	HR^a^	95% CI^a^	*p*
*Age group*				
< 2	102 (29%)	–	–	
≥ 2	248 (71%)	0.44	0.30, 0.64	< 0.001
Liver	127 (36%)	1.59	1.05, 2.42	0.03
Spleen	43 (12%)	1.55	0.86, 2.78	0.14
Hema	42 (12%)	1.08	0.6, 1.95	0.79
Lungs	24 (6.9%)	1.70	0.92, 3.13	0.09

^a^*HR* hazard ratio, *CI* confidence interval

Model: R^2^ = 0.139; c-index (standard error) = 0.684 (0.024); Obs/Exp ratio = 1.02

## Discussion

PPMLCH is a part of a clonal disease and has a different behavior from that of the adult form known to be reactionary to tobacco antigen [9, 10]. We retrospectively analyzed those PPMLCH patients presenting with typical chest high resolution CT nodules and cysts with or without clinical respiratory manifestations. With all the biases that could be manifested in retrospective studies, it remains a reference experience issued from the largest Egyptian center targeting such a population. Current publications define PPM LCH by the clinical respiratory manifestations and or the nodulo-cystic radiological pathognomonic lesions appearing on plain chest x ray or high resolution CT [27–30].

Ha et al. showed seldom clinical findings in LCH pediatrics with lung involvement [31]. Moreover, Grenier et al. and Vargas et al. showed that chest X-ray has a limited sensitivity and specificity than CT in detecting and characterizing early and subtle changes [32, 33]. Subsequently, the physician’s perception of the lung disease varies considerably between underestimation of mild radiologic lesions and thus restricting the diagnosis to only diffuse bilateral typical nodular/cystic lesions [18, 28] to overestimation by retaining nonspecific ones. These could include radiologic ground glass opacity, cord shadows, patches and thymic enlargement [19]. This renders stratification, somewhat uncertain with subsequent inaccurate management. Admitting the unreliability of clinical respiratory manifestations and X-ray to diagnose pulmonary involvement, we depended on low dose high resolution CT chest. This attitude favored the inclusion of patients with the least nodular lesions to diagnose pulmonary LCH in order to adjust risk stratification. Interestingly, the chest x ray proved to have a positivity in nearly 25% in relation to CT in our population, denoting a less reliable sensitive tool. CT reveals progressive sequential abnormality starting by single nodule to cavitary nodule, thick then thin-walled cyst and finally confluent cysts [34]. The diagnosis of lung lesion involvement remains subjective with variable incidences ranging between 10 and 50% of children with MS LCH [8, 18, 19]. We found that 19% of the population under systemic treatment had an association between the lung and any other organ. In the absence of CT, we would have misdiagnosed lung involvement and down stratified multisystem RO− to single system in 10 patients representing 15% of our whole population. Such a subgroup could have received shorter treatment with subsequent possible reactivation. Interestingly, by retaining patients with minimum nodules, our incidence did not exceed what has been reported elsewhere. Our cohort represented 16% of the whole LCH population, like the international data around 15% of LCH patients [5]. In our cohort of 67 patients with lung involvement, the OS and EFS were statistically significant lesser when lung was associated with RO+ rather than RO−. This was confirmed when assessing the association according to sub risk stratification, where the OS and EFS were lesser when the lung was associated to MSRO+ in relation to each MSRO−, USMF and USUF. Other studies are concordant with our results where Ronceray et al. showed that the lung is not a high-risk organ thus not an independent cause for mortality [8]. This is confirmed by lesser 5-year OS and EFS with lung when associated to RO+ [19] and to both hematologic and hepatic involvement rather than with each one alone [35]. The role of increased lung lesion severity on clinical radiological basis to affect the outcome is unclear in the literature. In one study, Bano et al. showed that death to respiratory failure was an exception suggesting the role of other RO+ involvement, rather than the lung specifically [18]. Contrarily, in a retrospective national cohort study, the French LCH group estimated the role of severe clinical lung involvement in intensive care unit admission and high mortality [28]. This study group criteria of lung involvement depended clinically on dyspnea, cough, cyanosis, while radiologically on symmetric, bilateral reticulonodular opacities, and a scoring system evaluating separately a combination of CT scan nodules [36]. In our study, for expressing lung lesions severity as a risk factor, we included clinical respiratory manifestations with its variable stages [25], and we recommended a CT radiological triad criteria: bilateral, diffuse taking the whole lung field, and extensive with innumerable lung lesions with or without pneumothorax. This triad of radiological lung lesions severity, is relatively an objective simplified descriptive tool for assessment in relation to the detailed lobar assessment of the scoring system in the French study [36]. In our study, in the 67 patients, diverse criteria of lung lesions severity including clinical severe respiratory manifestations, radiological diffuse and extensive lesions, presence of CXR changes were all associated with statistically significant lesser EFS. When comparing the lung population with non-lung one, out of 350 LCH patients receiving systemic treatment, the radiological triad of increased lung lesions severity was associated with a statistically significant lesser OS and EFS. At first glance this could be related to associated high-risk organs; emphathized by a statistically significant lesser OS and EFS with hepatic involvement and a lesser EFS with splenomegaly. However, there was also a statistically significant lesser EFS with the radiological triad of increased lung lesions severity in the RO− group (excluding risk organs). What is peculiar in our observation is that it takes into consideration not only the lung involvement but also its degree of severity in affecting the outcome. Moreover, it raises the question about the risk group/lung lesions severity interrelation. By univariate analysis, we could reach statistically significant results of increased lung lesions severity specific variables on survival. However, in our adjusted model in multivariate analysis, age group < 2 years old and liver involvement showed important positive prognostic factor. Although severe lung involvement did not retain statistical significance at alpha of 0.05, it had a large effect size (HR = 1.7) and the p-value was small 0.09 denoting a tendency to be significant. The predictive potential of severe lung involvement warrants external validation with larger samples. These results are concordant with those of Ronceray et al. [8], at least as regard the liver involvement. Lung involvement although it did not influence survival by their cox regression multivariate analysis, the lung lesion severity was not taken into consideration as in our experience.

We agree it is still a retrospective study with limited numbers in relation to the presence of many possible confounders. Of them, the use of 2 successive protocols. However, intermediate dose methotrexate for RO+ in LCH III and omitted in LCH IV did not to affect the outcome in those patients [4]. Disease reactivation on a lung mode in those 283 patients of non-lung population could not be assessed due to limited numbers and it would be a point of research in a further study. However, our research remains a trial to clarify the role of lung lesions severity in the outcome of PPMLCH. The fate of lung lesions was variable between RO− and RO+ . Solitary lesions, present more in RO−, were mostly of favorable outcome and cleared at last follow up. Contrarily to severe lung lesions present more in RO+ group and either were progressive or stationary at last follow up. In general, lung lesions in RO− group had no impact on OS, but was responsible of more failure to treatment and thus a lesser EFS. Otherwise, other lesions present in RO+ could be associated to lethal outcome. In this cohort, 8 patients -all in the RO+ groups- died. The lung was responsible exclusively of death in 2 of them. While the remaining 6 deaths were due to multisystem failure. This shows that lung lesions could be properly evaluated and linked to an appropriate treatment as showed by others [37–39].

## Conclusion

High resolution CT chest is helpful to accurately stratifying the pulmonary LCH patient’s disease. Although considered a RO-, increased lung lesions severity, either clinically or radiologically might be associated with a lesser EFS survival. This is demonstrated by statistically significant univariate analysis and a tendency to be significant cox regression multivariate analysis. Moreover, the impact of lung lesions severity on lesser EFS proved to be statistically significant in the RO− group. This deserves further efforts in targeting such a population for treatment adjustment. Otherwise, in the RO+ group, whether mortality is related to other risk organs or the lung lesions severity itself, the interrelation is suggested to be investigated with further studies.

### Supplementary Information

**Additional file 1**. The details of clinical radiological lung characteristics and outcome.

## Data Availability

The data that support the findings of this study are not openly available due to reasons of sensitivity and are available from the corresponding author upon reasonable request.

## Availability of data and materials

Through electronic medical records “Cerner” and the datasets used/analyzed during this study are available from the corresponding author on request.
