# Supplementary material for: Pediatric pulmonary multisystem langerhans cell histiocytosis: does lung lesion severity affect the outcome?
Source: Orphanet J Rare Dis. 2023 Nov 17;18:361. doi: 10.1186/s13023-023-02970-5 (PMC10655418; doi:10.1186/s13023-023-02970-5)
Supplement: Supplementary file 1 — Additional file 1. The details of clinical radiological lung characteristics and outcome. [file 13023_2023_2970_MOESM1_ESM.docx]

Table S1: The details of clinic radiological lung characteristics and outcome

| **UPN*** | **G** | **Age years** | ***ASS Org** | **RM** | **X ray** | **CT** | **Dist** | **Extent** | **Lat** | **severe** | **CXR changes** | **Response induction **** | **DP** | **REA** | **Response**  **LFU ***** | **DIED** | **Cause of death** |
| --- | --- | --- | --- | --- | --- | --- | --- | --- | --- | --- | --- | --- | --- | --- | --- | --- | --- |
| 1* | F | <=2 | MSRO+ | RDIV | Bilat nodules | Multi bilat. Nodulo cysts | Diff | Ext | Bilat | S | + | C | N | N | C | No | No |
| 2* | M | >2 | MSRO+ | No | Clear Rt lung fields | Multi bilat tiny nodular | Diff | Non Ext | Bilat | NS | - | C | N | Y | Prog | Yes | DP (MSF) |
| 3* | M | <=2 | MSRO+ | No | Multi bilat basal nodular | Diff bilat reticulonodular | Diff | Non Ext | Bilat | NS | + | C | N | Y | C | No | No |
| 4* | M | <=2 | MSRO+ | No | Clear both lung fields | Bilat. reticulonodular cyst | Diff | Ext | Bilat | S | - | P | N | Y | Prog | Yes | Pulm prog |
| 5* | M | >2 | MSRO+ | RDIII | Bilat diffuse reticular | Bilat.diff. micronodulations | Diff | Ext | Bilat | S | + | P | Y | N | Prog | Yes | DP (MSF) |
| 6* | F | <=2 | MSRO+ | No | Clear both lung fields | Bilat.nodular densities | L | Non Ext | Bilat | NS | - | C | N | N | C | No | No |
| 7 | M | <=2 | MSRO+ | RDIII | Multi reticulo nodular. | Diff. bilat. reticulonodular | Diff | Ext | Bilat | S | + | Stat | Y | N | Stat | Yes | DP (MSF) |
| 8 | M | <=2 | MSRO+ | No | Bilat. Nodules | Bilat. variable sized nodules | Diff | Ext | Bilat | S | + | R | N | N | C | No | No |
| 9 | M | <=2 | MSRO+ | No | Bilat reticulo-nodular | Bilat. Reticulonodular | Diff | Non Ext | Bilat | NS | + | C | N | NE | C | NE | NE |
| 10 | M | >2 | MSRO+ | No | Diff. reticulo-nodular | Bilat nodular cysts. | Diff | Ext | Bilat | S | + | Stat | N | N | C | No | No |
| 11 | M | <=2 | MSRO+ | No | Clear both lung fields | Bilat. Reticulonodular | Diff | Ext | Bilat | S | - | R | Y | N | C | No | No |
| 12 | F | >2 | MSRO+ | No | Clear Rt lung fields | Lt lower reticulo-nodular | L | Non Ext | U | NS | - | R | N | N | C | No | No |
| 13 | F | <=2 | MSRO+ | No | Clear both lung fields | Right lobe single nodule | L | Non Ext | U | NS | - | P | N | Y | R | Yes | DP (MSF) |
| 14 | M | <=2 | MSRO+ | RDIII | Clear both lung fields | Bilat. upp reticulo-nodular | L | Non Ext | Bilat | NS | - | Stat | N | Y | R | LFU | LFU |
| 15 | M | <=2 | MSRO+ | No | Clear both lung fields | Bilat. tiny nodules | Diff | Ext | Bilat | S | - | C | N | N | C | No | No |
| 16 | F | <=2 | MSRO+ | No | Clear both lung fields | Faint Rt middle lobe nodule | L | Non Ext | U | NS | - | P | Y | N | C | No | No |
| 17 | F | <=2 | MSRO+ | RDII | Clear right lung fields. | Bilateral tiny fibro nodules, | Diff | Ext | Bilat | S | - | R | Y | N | R | Yes | DP (MSF) |
| 18 | F | <=2 | MSRO+ | No | Clear both lung fields | Bilat. Mult-reticulonodular | Diff | Ext | Bilat | S | - | NE | Y | N | NE | Yes | DP (MSF) |
| 19 | M | <=2 | MSRO+ | No | Diff. reticulo nodular cysts | Bilat. Diff. reticulonodcysts, | Diff | Ext | Bilat | S | + | P | Y | N | Prog | Yes | Pulm prog |
| 20 | M | >2 | MSRO+ | No | Clear both lung fields | Rt basal reticulonodular | L | Ext | Bilat | NS | - | C | N | N | C | No | No |
| 21 | M | >2 | USMF | No | Clear both lung fields | Tiny nodular right lower | L | Non Ext | U | NS | - | R | N | Y | C | No | No |
| 22 | M | >2 | USMF | No | Clear both lung fields | Solitary Rt upper nodule | L | Non Ext | U | NS | - | Stat | N | N | C | No | No |
| 23 | F | >2 | USMF | No | Clear both lung fields | Bilat. Few scatter nodules | L | Non Ext | Bilat | NS | - | Stat | N | N | C | No | No |
| 24 | M | >2 | USMF | No | Clear both lung fields | 2 right mid lobe nodules | L | Non Ext | U | NS | - | C | N | Y | C | No | No |
| 25 | F | >2 | USMF | No | Clear both lung fields | Subpleural nodules. | L | Non Ext | U | NS | - | R | N | N | C | No | No |
| 26 | M | >2 | USMF | No | Clear both lung fields | few bilateral nodules | L | Non Ext | U | NS | - | Stat | N | N | R | No | No |
| 27 | M | >2 | USUF | No | Clear both lung fields | Small bilat nodules | L | Non Ext | Bilat | NS | - | R | N | N | C | No | No |
| 28 | F | >2 | USUF | No | Clear both lung fields. | multi tiny right nodules | L | Non Ext | U | NS | - | C | N | N | C | No | No |
| 29 | M | >2 | USUF | No | Clear both lung fields | subpleural basal nodule | L | Non Ext | U | NS | - | Stat | N | N | Stat | No | No |
| 30 | F | >2 | USUF | No | Clear both lung fields | Subpleural lingual nodule | L | Non Ext | U | NS | - | C | N | N | C | No | No |
| 31* | M | <=2 | MSRO- | No | Bilat Fibro nodular | Bilat Multi pneumatoceles | Diff | Ext | Bilat | S | + | C | N | Y | C | No | No |
| 32 | F | >2 | MSRO- | No | Clear both lung fields | Bilat Few small nodules | L | Non Ext | Bilat | NS | - | Stat | N | N | stat | No | No |
| 33 | M | >2 | MSRO- | No | Clear both lung fields | Lt Two tiny nodules | L | Non Ext | U | NS | - | C | N | N | C | No | No |
| 34 | M | >2 | MSRO- | No | Clear both lung fields | Bilat tiny nodules | L | Non Ext | Bilat | NS | - | C | N | N | C | No | No |
| 35 | M | >2 | MSRO- | No | Clear both lung fields | few tiny bilat. Nodules | Diff | Non Ext | Bilat | NS | - | C | N | N | C | No | No |
| 36 | F | <=2 | MSRO- | No | Clear both lung fields | Small bilateral nodules | Diff | Non Ext | Bilat | NS | - | Stat | N | N | C | No | No |
| 37 | F | >2 | MSRO- | No | Clear both lung fields | RT reticulo-nodular. | Diff | Non Ext | Bilat | NS | - | C | N | Y | C | No | No |
| 38 | M | >2 | MSRO- | No | Clear both lung fields | RT nodule | L | Non Ext | U | NS | - | Stat | N | N | stat | No | No |
| 39 | M | >2 | MSRO- | No | Bilat. reticulo-nodular | Bilat. reteiculo-nodular | Diff | Ext | Bilat | S | + | C | N | Y | C | No | No |
| 40 | F | >2 | MSRO- | No | Clear both lung fields | Muti Bilat nodules | Diff | Ext | Bilat | S | - | C | N | N | C | No | No |
| 41 | M | <=2 | MSRO- | No | Clear both lung fields | left apical nodule | L | Non Ext | U | NS | - | Stat | N | N | C | No | No |
| 42 | F | >2 | MSRO- | RDIII | Bilateral reticulocystic | Bilat.pneumothorax,cysts | Diff | Ext | Bilat | S | + | R | Y | N | stat | No | No |
| 43 | M | <=2 | MSRO- | No | Clear both lung fields | Lt Apex lower lobe bulla | Diff | Ext | U | NS | - | C | N | N | C | No | No |
| 44 | M | <=2 | MSRO- | No | Clear both lung fields | Rt apex, Rt reticulonodular | Diff | Non Ext | U | NS | - | P | N | N | C | No | No |
| 45 | M | >2 | MSRO- | No | Clear both lung fields | Tiny Rt nodule | Diff | Non Ext | U | NS | - | Stat | N | N | stat | No | No |
| 46^$^ | M | >2 | MSRO- | RDIII | Bilat nodules, Lt PN thorax | Left pneumothorax, Rt cysts | Diff | Ext | Bilat | S | + | R | Y | N | R | No | No |
| 47 | M | >2 | MSRO- | No | Clear both lung fields | Tiny right sided nodule | L | Non Ext | U | NS | - | C | N | N | C | No | No |
| 48 | F | >2 | MSRO- | RDII | Lt upper lung zone | bilateral extensive diff. cysts | Diff | Ext | Bilat | S | + | Stat | N | N | stat | No | No |
| 49 | F | >2 | MSRO- | No | Clear both lung fields | Multi bilat nodules | Diff | Non Ext | Bilat | NS | - | C | N | N | C | No | No |
| 50 | F | >2 | MSRO- | No | Clear both lung fields | Rt lower lobe nodule | L | Non Ext | U | NS | - | Stat | N | N | C | No | No |
| 51 | M | >2 | MSRO- | No | Bilat patchy opacities | Bilat. reticulo-nodular | Diff | Ext | Bilat | S | + | R | N | N | stat | No | No |
| 52 | M | >2 | MSRO- | No | Clear both lung fields | Small right basal nodule | L | Non Ext | U | NS | - | C | N | Y | C | No | No |
| 53 | F | <=2 | MSRO- | No | Clear both lung fields | Bilat. Diff. reticular nodular | Diff | Ext | Bilat | S | - | NE | N | N | NE | No | No |
| 54 | M | >2 | MSRO- | No | Clear both lung fields | Rt. nodule, left cystic basal | Diff | Non Ext | Bilat | NS | - | C | N | N | C | No | No |
| 55 | M | >2 | MSRO- | No | Clear both lung fields | Lt lower tiny nodule | L | Non Ext | Bilat | NS | - | C | N | N | C | No | No |
| 56 | M | >2 | MSRO- | No | Clear both lung fields | Rt. tiny peripheral nodule | L | Non Ext | U | NS | - | Stat | N | N | Stat | No | No |
| 57 | M | <=2 | MSRO- | No | Upper Bilat. nodular | Bilat diff. tiny nodulo cystic | Diff | Ext | Bilat | S | + | R | N | Y | C | No | No |
| 58 | M | >2 | MSRO- | No | Clear both lung fields | Lt multi scattered cysts | Diff | Ext | Bilat | S | - | P | Y | N | R | No | No |
| 59 | F | >2 | MSRO- | No | Clear both lung fields | Right basal small nodule | Diff | Non Ext | U | NS | - | C | N | Y | C | No | No |
| 60 | F | <=2 | MSRO- | No | Clear both lung fields | Reticulo nodular infiltration | Diff | Ext | Bilat | S | - | P | N | N | R | No | No |
| 61 | F | >2 | MSRO- | No | Clear both lung fields | Rt lower lobe nodule | L | Non Ext | Bilat | NS | - | Stat | N | N | C | No | No |
| 62 | M | >2 | MSRO- | No | Clear both lung fields | Uni Rt lung nodule | L | Non Ext | U | NS | - | R | N | N | R | No | No |
| 63 | M | >2 | MSRO- | No | Clear both lung fields | Bilat. reticulo nodular | L | Non Ext | Bilat | NS | - | R | N | N | C | No | No |
| 64 | F | <=2 | MSRO- | No | Clear both lung fields | Rt few subpleural nodules | Diff | Non Ext | Bilat | NS | - | P | N | N | R | No | No |
| 65 | F | >2 | MSRO- | No | Clear both lung fields | Bilateral few nodules | Diff | Non Ext | Bilat | NS | - | R | N | N | Stat | No | No |
| 66 | M | >2 | MSRO- | No | Clear both lung fields | Bilateral cystic lesions | Diff | Ext | Bilat | S | - | P | N | N | Stat | No | No |
| 67 | F | >2 | MSRO- | No | Clear both lung fields | Disperse pulmonary bullae | Diff | Ext | Bilat | S | - | Stat | N | N | Stat | No | No |

**ASS** Org risk organ association, **Bilat:** Bilateral, **C:** Cleared, CP costophrenic, CXR chest X ray, **Diff:** Diffuse, **Dist**: Distribution, **DP**: Disease Progression, **Ext:** Extensive, **F:** female, **G**: Gender, **Lat**: Laterality **L:** Localized,  **Lt** left, **LFU:** lost Follow Up, **M:** Male, **MSRO+** multisystem high risk organ, **MSRO**- multisystem low risk organ **MSF:** Multi System failure, **N** No, **Non Ext:** Non Extensive, **RO-:** Non Risk Organ, **NE:** not evaluable, **Non ext** non extensive, **NS:** Not Severe, **PN** pneumothorax, **P:** progressed, **Pulm Prog** Pulmonary progression, **Rt** right, **Radio Resp end ttt** Radiological response after end of treatment, **Radio Resp Ind** Radiological response post induction, **REA**: Reactivation , **R:** regressed, **RDI:** Respiratory Distress grade I **RDII:** Respiratory Distress grade II **RDIII:** Respiratory Distress grade III , Respiratory Distress grade IV, RM: Respiratory manifestations **RO+:** high Risk Organ, **S**: severe, **Stat**: stationary , **USMF**: Uni system multi focal , **USUF**: Uni system uni focal, **U:** Unilateral, **UPN** Unique patient number,  **Y** yes

*Lung being high risk organ associated to MS RO+ (UPN 1-6) and to MS RO- (UPN 31) all treated with LCH III protocol from 2007 to 2011 .Otherwise, starting from 2012 lung considered a low risk organ associated to USUF or USMF or MSRO- or MSRO+ and treated with LCHIV IV protocol.

** Lung lesions Response to induction whether cleared, regressed, stationary, progressed or non-evaluable

*** Lung lesions Response at last follow up whether cleared, regressed, stationary, progressed or non-evaluable

$ UPN 46 Suprasellar brain space occupying tumorous lesion associated with Lung severe lesions of favorable outcome with salvage 2 chloredeoxyadenosine 2 CdA
